# The utilisation of ctDNA approaches for residual disease detection during neoadjuvant and perioperative immunotherapy in oesophagogastric cancers

**DOI:** 10.1002/ctm2.70223

**Published:** 2025-02-04

**Authors:** Ronan J. Kelly, Valsamo Anagnostou, Vincent K. Lam, Ali H. Zaidi

**Affiliations:** ^1^ The Charles A. Sammons Cancer Center Baylor University Medical Center Dallas Texas USA; ^2^ The Sidney Kimmel Comprehensive Cancer Center Johns Hopkins University Baltimore Maryland USA; ^3^ Allegheny Health Network Cancer Institute Allegheny Health Network Pittsburgh Pennsylvania USA

**Keywords:** ctDNA, MRD, oesophageal cancer, precision medicine

1

The optimal management of operable oesophageal/GEJ (E/GEJ) cancer has been the subject of much debate with the traditional trimodality approach of chemoradiotherapy followed by surgery as established by the CROSS trial^1^ in 2012 being challenged by the recently presented results of the ESOPEC trial^2^ which demonstrated superiority of the perioperative fluorouracil, leucovorin, oxaliplatin and docetaxel (FLOT) chemotherapy regimen. Unfortunately, ESOPEC a phase III German led study that enrolled patients between 2016 and 2020 did not compare FLOT to the other standard of care which is chemoradiation followed by adjuvant nivolumab. CheckMate 577 a phase III international adjuvant study^3^ published in 2021 investigated the efficacy of the PD‐1 inhibitor nivolumab as a systemic agent in an attempt to overcome the challenges posed by utilising radiation sensitising low‐dose chemotherapy used in the CROSS regimen. CheckMate 577 demonstrated a doubling in median disease‐free survival (mDFS) from 11.0 to 22.4 months (HR 0.69) with the use of adjuvant nivolumab in tumours that had failed to attain a pathological complete response (pCR) post trimodality therapy. mDFS was the primary endpoint of the study but interestingly the secondary endpoint of median distant metastasis‐free survival was also increased from 17.6 to 28.3 months (HR 0.74), indicating a systemic effect for PD‐1 inhibition above and beyond loco‐regional benefits. As is the norm in large adjuvant studies, overall survival (OS) has not been reported as yet requiring a number of years to meet predefined events but challenges in interpretation will exist given the widespread use of immune checkpoint inhibitors (ICIs) in the metastatic setting.^4^ The question therefore remains which is a better approach in operable E/GEJ cancers—perioperative FLOT or trimodality therapy followed by adjuvant nivolumab? In 2025, it may be the wrong question to ask whether chemotherapy or radiation is better, as the answer will vary depending on the biology of an individual's tumour. With the use of precision medicine, we would hope to be able to gain a more nuanced understanding and define an optimal way to select the most appropriate therapeutic options. This approach aims to ensure that patients achieve the best possible results while avoiding over‐ or under‐treatment and undue toxicities.

The use of circulating tumour DNA (ctDNA) in detecting and tracking minimal residual disease (MRD) may improve upon the use of traditional ypTNM staging and pCR as surrogates for long‐term survival.

In our study published in *Nature Medicine* in April 2024,^5^ we sought to measure systemic tumour burden kinetics longitudinally using a tumour‐agnostic, matched WBC DNA‐informed deep sequencing approach coupled with a branched logic to assign variant cellular origin in the pre‐ and post‐operative setting as a more accurate early determinant of therapeutic efficacy. We defined ctDNA positivity if ≥ 1 tumour‐derived variants were detected at any mutant allele frequency, whereas ctDNA was deemed undetectable if no tumour‐derived variants were detected at the specified timepoint. To the best of our knowledge, our study is one of the first to assess MRD in E/GEJ cancer patients treated with neoadjuvant immunotherapy combined with chemoradiotherapy. Notably, when evaluating ctDNA levels at various timepoints in the perioperative period (baseline, after ICI induction, preop and postop), the presence or absence of ctDNA was strongly predictive of clinical outcomes. We found that patients with undetectable ctDNA post 2 cycles of induction ICI alone had a significantly longer relapse free survival (RFS). These findings suggest that a proportion of patients, even those without defects in the DNA mismatch repair machinery, may benefit from neoadjuvant immunotherapy alone with the possibility of avoiding chemotherapy and or radiation with clearance of ctDNA after 2–4 weeks being an early predictor of favourable outcomes. This strategy should be evaluated in future studies. In the perioperative period, patients with ctDNA negative disease pre‐operatively had a longer RFS compared to those with detectable ctDNA. Post‐operatively (3–12 weeks), patients that were ctDNA MRD negative attained a longer RFS compared to patients that were MRD positive.

Taken together, our findings suggest that ctDNA status as early as the first month into treatment in the neoadjuvant window may be critical in enriching for the patients most likely to benefit from specific perioperative regimens and inform treatment escalation or de‐escalation decisions, ultimately maximising therapeutic benefit. While pCR has historically been used as an early indicator of therapeutic efficacy in the neoadjuvant ICI setting, we are increasingly recognising the clinical and biological heterogeneity of tumours with non‐pCR. Notably, ctDNA status accurately distinguished non‐pCR patients with differential clinical outcomes. Interestingly, none of the patients within the non‐pCR group that were ctDNA negative after ICI induction recurred compared to patients within the same non‐pCR group that were ctDNA positive after ICI. These findings, while requiring further validation, suggest that clinical decision making for peri‐operative management may be best informed by ctDNA molecular kinetics rather than pathological responses. Linking circulating tumour burden changes with functional anti‐tumour immune responses, neoantigen‐specific T cell responses were a mirror reflection of ctDNA kinetics, such that ctDNA clearance was associated with the detection and expansion of neoantigen‐specific T cells in patients attaining longer RFS and OS. These findings further support the value of ctDNA analyses in capturing functional anti‐tumour immune responses and may allow us to optimally design future neoadjuvant or perioperative studies utilising combined blood‐based assays to escalate or de‐escalate treatment in an attempt to maximise response.

Collectively, our results suggest that monitoring systemic tumour burden kinetics during neoadjuvant ICI and into the post‐operative period may enhance precision in the clinical management of patients with E/GEJ cancer and provide an opportunity for us to embrace molecular approaches to move beyond simply asking whether chemotherapy is better than radiation in this challenging tumour type that has seen limited advances over many decades.

## AUTHOR CONTRIBUTIONS

RJK, AZ, VL and VA conceived the study, contributed to the study design, performed the clinical data statistical analysis. contributed to data analysis, interpretation and writing. All authors contributed to data interpretation and edited the manuscript.

## CONFLICT OF INTEREST STATEMENT

**Ronan J. Kelly** reports receiving advisory board/consulting fees from Amgen, Astellas, AstraZeneca, Beigene, Bristol Myers Squibb, Cardinal Health, Daiichi Sankyo, Eisai, Eli Lilly, EMD Serono, Exact Sciences, Grail, Illumina, Ipsen, Merck, Novartis, Novocure, OncoHost, Phillips, Takeda, Toray and grant support paid to Johns Hopkins University and Baylor University Medical Center from Bristol Myers Squibb and Eli Lilly. **Valsamo Anagnostou** receives research funding to Johns Hopkins University from Astra Zeneca and Personal Genome Diagnostics, has received research funding to Johns Hopkins University from Bristol‐Myers Squibb and Delfi Diagnostics in the past 5 years, is an advisory board member for AstraZeneca and Neogenomics (compensated) and receives honoraria from Foundation Medicine, Guardant Health and Labcorp/Personal Genome Diagnostics. **Valsamo Anagnostou** is an inventor on patent applications (63/276,525, 17/779,936, 16/312,152, 16/341,862, 17/047,006 and 17/598,690) submitted by Johns Hopkins University related to cancer genomic analyses, ctDNA therapeutic response monitoring and immunogenomic features of response to immunotherapy that have been licensed to one or more entities. **Vincent K. Lam** has served in a consultant/advisory role for Pfizer, Genentech/Roche, Iovance Biotherapeutics, Anheart Therapeutics, Takeda, Seattle Genetics, Bristol Myers Squibb, AstraZeneca and Guardant Health, and has received research funding from GlaxoSmithKline, Bristol Myers Squibb, AstraZeneca, Merck and Seattle Genetics. **Ali H. Zaidi** is serving in a consultant/advisory role for Previse, Prognomiq, Gilead, Delfi Diagnostics and BilliontoOne. He has received significant research funding from Eli Lilly, Prognomiq, BilliontoOne, Genece Health, Roche, Myriad Genetics, Delfi Diagnostics and Tempus. **Ali H. Zaidi** has equity interest in Previse, Tg Therapeutics and Gritstone Bio.

## ETHICS STATEMENT

The clinical trial adhered to the ethical principles outlined in the Declaration of Helsinki.
